# Bile Acid-Mediated Sphingosine-1-Phosphate Receptor 2 Signaling Promotes Neuroinflammation during Hepatic Encephalopathy in Mice

**DOI:** 10.3389/fncel.2017.00191

**Published:** 2017-07-05

**Authors:** Matthew McMillin, Gabriel Frampton, Stephanie Grant, Shamyal Khan, Juan Diocares, Anca Petrescu, Amy Wyatt, Jessica Kain, Brandi Jefferson, Sharon DeMorrow

**Affiliations:** ^1^Department of Research, Central Texas Veterans Health Care SystemTemple, TX, United States; ^2^Department of Internal Medicine, College of Medicine, Texas A&M University Health Science CenterTemple, TX, United States; ^3^Department of Internal Medicine, Baylor Scott & White HealthTemple, TX, United States

**Keywords:** microglia, cytokines, CCL2, acute liver failure, neurons, S1PR2

## Abstract

Hepatic encephalopathy (HE) is a neuropsychiatric complication that occurs due to deteriorating hepatic function and this syndrome influences patient quality of life, clinical management strategies and survival. During acute liver failure, circulating bile acids increase due to a disruption of the enterohepatic circulation. We previously identified that bile acid-mediated signaling occurs in the brain during HE and contributes to cognitive impairment. However, the influences of bile acids and their downstream signaling pathways on HE-induced neuroinflammation have not been assessed. Conjugated bile acids, such as taurocholic acid (TCA), can activate sphingosine-1-phosphate receptor 2 (S1PR2), which has been shown to promote immune cell infiltration and inflammation in other models. The current study aimed to assess the role of bile-acid mediated S1PR2 signaling in neuroinflammation and disease progression during azoxymethane (AOM)-induced HE in mice. Our findings demonstrate a temporal increase of bile acids in the cortex during AOM-induced HE and identified that cortical bile acids were elevated as an early event in this model. In order to classify the specific bile acids that were elevated during HE, a metabolic screen was performed and this assay identified that TCA was increased in the serum and cortex during AOM-induced HE. To reduce bile acid concentrations in the brain, mice were fed a diet supplemented with cholestyramine, which alleviated neuroinflammation by reducing proinflammatory cytokine expression in the cortex compared to the control diet-fed AOM-treated mice. S1PR2 was expressed primarily in neurons and TCA treatment increased chemokine ligand 2 mRNA expression in these cells. The infusion of JTE-013, a S1PR2 antagonist, into the lateral ventricle prior to AOM injection protected against neurological decline and reduced neuroinflammation compared to DMSO-infused AOM-treated mice. Together, this identifies that reducing bile acid levels or S1PR2 signaling are potential therapeutic strategies for the management of HE.

## Introduction

Hepatic encephalopathy (HE) is a neuropsychiatric complication that occurs due to deteriorating hepatic function and this syndrome influences patient quality of life, clinical management strategies and survival (Butterworth, 2011). Patients with acute liver failure that develop HE have a 70% 5 year survival if they undergo liver transplantation though only one in five patients with acute liver failure qualify and receive a transplant, indicating a substantial need to identify novel treatment strategies for this patient population (Bernal et al., 2013; Reuben et al., 2016). The pathogenesis of HE is not fully understood with the current views focusing on the elevations of circulating and brain ammonia, increased oxidative stress and inflammation that contribute to its progression (Aldridge et al., 2015).

When microglia become activated in the brain, they begin producing proinflammatory cytokines and factors that promote oxidative stress (Voloboueva and Giffard, 2011). Due to both neuroinflammation and oxidative stress contributing to the pathogenesis of HE, it is not surprising that microglia activation has been observed during HE in animal models (Jiang et al., 2009a,b; Rodrigo et al., 2010; Chastre et al., 2012; Rangroo Thrane et al., 2012; Dadsetan et al., 2016; McMillin et al., 2016b) in patients with HE (Wright et al., 2007; Butterworth, 2011; Dennis et al., 2014), as well as in a rat model of hyperammonemia (Hernández-Rabaza et al., 2016). The mechanisms that lead to the activation of microglia during HE are not fully understood but we have shown that this can occur as a consequence of chemokine ligand 2 (CCL2) release from neurons acting on and activating microglia (McMillin et al., 2014a). However, the identification of factors that lead to the release of CCL2, and the subsequent neuroinflammation and neurological decline that result, are yet to be identified.

Bile acids are the amphipathic end products of cholesterol metabolism that contribute to hepatic, intestinal and metabolic disorders. In normal conditions bile acids are secreted into the intestine and 90% are reabsorbed back into the liver via the enterohepatic circulation (Dawson et al., 2009). During acute liver failure and chronic liver disease, the transport of bile acids from the hepatic sinusoids and into hepatocytes is disrupted and bile acid concentrations become significantly elevated in the serum (Neale et al., 1971; Pazzi et al., 2002). Elevated levels of bile acids in the brain have been reported in patients with HE due to acute liver failure and in a mouse model of acute liver failure elevated bile acids in the brain contribute to neurological decline (Bron et al., 1977; McMillin et al., 2016a). At this time the exact mechanisms that bile acids contribute to pathogenesis during HE are not known.

Conjugated bile acids, such as taurocholic acid (TCA), have been shown to activate sphingosine 1-phosphate receptor 2 (S1PR2) in hepatocytes and the activation of ERK1/2 and AKT, downstream signaling mediators of S1PR2-mediated signaling, were inhibited by the S1PR2 antagonist JTE-013 (Studer et al., 2012). During cholestatic liver injury, where there is a significant increase of hepatic bile acid concentrations, intraperitoneal injection of JTE-013 reduced immune cell infiltration and interleukin-6 (IL-6), tumor necrosis factor alpha (TNFα) and CCL2 expression in the liver (Yang et al., 2015). In the brain S1PR2 is expressed primarily in the gray matter with cortical neurons, hippocampal pyramidal cells and retinal ganglion cells expressing this receptor (Kempf et al., 2014).

At this time no data exist concerning the role of S1PR2-mediated signaling in the brain due to increased bile acid concentrations following acute liver failure. Therefore, the aim of this study was to classify bile acid-mediated S1PR2 signaling during HE and manipulate both bile acids and S1PR2 to determine their effects on neurological decline and neuroinflammation. This research may help identify if bile acid-mediated S1PR2 signaling could be a therapeutic target for management of neuroinflammation during HE due to acute liver failure.

## Materials and Methods

### Materials

The Total Bile Acid Assay was purchased from Diazyme Laboratories (Poway, CA, USA). Antibodies against IBA1 were purchased from Wako Chemicals USA (Richmond, VA, USA). The real-time PCR (RT-PCR) primers for CCL2, IL-6, S1PR2 and TNFα were purchased from SABiosciences (Frederick, MD, USA). CCL2, IL-6 and TNFα ELISAs were purchased from R&D systems (Minneapolis, MN, USA). JTE-013 was purchased from Tocris (Minneapolis, MN, USA). The cholestyramine and AIN-93G control diets were formulated and supplied by Dyets Inc. (Bethlehem, PA, USA). Cell culture media and supplements were purchased from Thermofisher Scientific (Waltham, MA, USA). All other assays and chemicals were purchased from Sigma-Aldrich (St. Louis, MO, USA) unless otherwise noted, and were of the highest grade available.

### AOM Model

Mouse *in vivo* experiments were performed using male C57Bl/6 mice (25–30 g; Charles River Laboratories, Wilmington, MA, USA). All animal experiments were approved by the Baylor Scott & White Health IACUC committee and were performed in accordance with the Animal Welfare Act and the Guide for the Care and Use of Laboratory Animals.

Acute liver failure and HE was induced via a single intraperitoneal injection of 100 μg/g of azoxymethane (AOM) into mice. After injection, mice were placed on heating pads set to 37°C to ensure they remained normothermic. Hydrogel and rodent chow were placed on cage floors to ensure access to food and hydration. After 12 h and every 4 h thereafter, mice were injected subcutaneously with 5% dextrose in 250 μL saline to ensure euglycemia and hydration. Following injection, mice were monitored at least every 2 h (starting at 12 h post AOM injection) for body temperature, weight and neurological score using previous published methodology (McMillin et al., 2014a,b; McMillin M. et al., 2015). Once neurological impairment was evident, mice were continuously monitored with formal assessments of temperature, body weight and neurological score performed each hour. The neurological score was assessed by an investigator blind to the treatments by assigning a score between 0 (absent) and 2 (intact) to each of the following parameters: the pinna reflex, corneal reflex, tail flexion, escape response, righting reflex and ataxia. The summation of these six reflexes gives a neurological score between 0 and 12. Tissue was collected prior to neurological symptoms (pre neurological), when minor ataxia and weakened reflexes were present (minor neurological), when major ataxia and deficits in reflexes were evident (major neurological) and at coma defined by a loss of righting and corneal reflexes.

In a subset of experiments mice were fed a diet containing cholestyramine (2%) or the control diet AIN-93G for 3 days prior to the injection of AOM. Specific inhibition of S1PR2 in the brain was achieved by infusing JTE-013 reconstituted in 50% DMSO directly into the lateral ventricle of the brain using Alzet brain infusion kits coupled to subcutaneous implanted minipumps (Cupertino, CA, USA) at 2 μg/kg/day for 3 days at coordinates AP -0.34, ML -1.0, DV -2.0 prior to AOM injection.

### Primary Neuron Isolation and Treatment

Primary neurons were isolated from P1 mouse pups from FVB/N mice or S1PR2 knockout mice (S1PR2^−/−^) using methodology previously described (McMillin et al., 2014b). Dr. Kazuaki Takabe from Virginia Commonwealth University School of Medicine provided the S1PR2^−/−^ mice used to generate pups for neuron cell isolations. Mouse pups were decapitated and whole brains were removed. Cortex was isolated and meninges and dura were removed. Cortical tissue was mechanically disrupted and filtered through a 100-μm filter. Neurons were pelleted by centrifugation at 1400 *g*. Neurons were resuspended in DMEM/F12 media containing 10% heat inactivated fetal bovine serum, 1% penicillin/streptomycin and 1% gentamycin and were plated on 12 well plates at 750,000 cells per well. After 24 h, the neurons were washed and media was replaced with Neurobasal^®^ media containing 2% B27 growth supplement, 1% penicillin/streptomycin and 1% gentamycin. After 10–12 days, neurons were treated with 10 μM TCA or 50 μM JTE-013. Cells were lysed and RNA was isolated for RT-PCR analyses.

### Brain Cell Isolation

Whole brains from adult C57Bl/6 mice were homogenized using the Miltenyi Biotec gentleMACS™ Dissociator (San Diego, CA, USA). Solutions used to ensure viability of cells were part of the Neural Tissue Dissociation Kit supplied from Miltenyi Biotec. Following dissociation into a single cell suspension, cells were passed through LS columns (Miltenyi Biotec) containing beads coated with CD11b antibodies (to isolate microglia) or GLAST antibodies (to isolate astrocytes) localized to the columns. The remaining cells not bound to the columns were kept as the neuron enriched fraction. LS columns were washed to remove the CD11b-bound cells or the GLAST-bound cells. The enriched cell fractions were then used for subsequent assays.

### Bile Acid Assay

Determination of total bile acid concentrations in the brain was performed as previously described (McMillin et al., 2016a). Vehicle and AOM-treated mice were euthanized and cortex tissue was dissected. Cortex homogenates were prepared by calculating the wet weight of brain tissue with subsequent homogenization in 100 mg/ml of ultrapure water using the Miltenyi Biotec gentleMACS™ Dissociator. Homogenates were spun down for 5 min at 16,100 *g* and supernatants were collected. Total bile acid content was assessed in these homogenates using the manufacturer’s instructions from Diazyme Laboratories. This kit was previously validated to effectively measure mouse total bile acids in cortex homogenates using this methodology (McMillin et al., 2016a).

### Metabolic Screen for Bile Acid Levels

Serum and cortex tissue were isolated from vehicle and AOM-treated mice at the pre-neurological and minor neurological states of decline and were sent to Metabolon (Morrisville, NC, USA) to assess certain bile acid levels as part of a larger metabolomics panel. Metabolon performed the assay and statistical analyses of these samples with *n* = 5 or greater per group.

### Immunofluorescence

Free-floating immunofluorescence staining was performed on 30 μm brain sections using anti-IBA1 immunoreactivity to detect morphology and relative staining of microglia. Immunoreactivity was visualized using fluorescent secondary antibodies labeled with Cy3 and counterstained with ProLong^©^ Gold Antifade Reagent containing 4′,6-diamidino-2-phenylindole (DAPI). Slides were viewed and imaged using a Leica TCS SP5-X inverted confocal microscope (Leica Microsystems, Buffalo Grove, IL, USA). Field fluorescence area of photomicrographs was determined by converting images to grayscale, inverting their color and quantifying field staining intensity with ImageJ software.

### Gene Expression Analyses

RNA was extracted from tissue, primary neurons or isolated cell fractions using an RNeasy Mini Kit (Qiagen, Germantown, MD, USA) according to the manufacturer’s instructions. Synthesis of cDNA was accomplished using a Bio-Rad iScript™ cDNA Synthesis Kit (Hercules, CA, USA). RT-PCR was performed as previously described (Frampton et al., 2012) using commercially available primers designed against mouse IL-6, CCL2, TNFα, S1PR2 and glyceraldehyde 3-phosphate dehydrogenase (SABioscience, Frederick, MD, USA). A ΔΔCT analysis was performed using vehicle-treated tissue or untreated primary neurons as controls for subsequent experiments (Livak and Schmittgen, 2001; DeMorrow et al., 2008).

### Quantitative Protein Assessments

Cortex tissue from all treatment groups was homogenized using a Miltenyi Biotec gentleMACS™ Dissociator and total protein was quantified using a ThermoFisher Pierce™ BCA Protein Assay kit. Capture antibodies against CCL2, IL-6, or TNFα were incubated overnight in 96-well plates. Each ELISA was performed according to the instructions provided from R&D Systems and the total input protein for each sample was 100 μg or serum diluted 1:10. Absorbance was read using a SpectraMax^®^ M5 plate reader from Molecular Devices (Sunnyvale, CA, USA). Data were reported as CCL2, IL-6 or TNFα concentration per mg of total lysate protein or per ml of serum.

### Liver Histology and Serum Chemistry

Paraffin-embedded livers were cut into 4 μm sections and mounted onto positively charged slides (VWR, Radnor, PA, USA). Slides were deparaffinized and stained with Hematoxylin QS (Vector Laboratories, Burlingame, CA, USA) for 1 min followed by staining for 1 min with eosin Y (Amresco, Solon, OH, USA) and rinsed in 95% ethanol. The slides were then dipped into 100% ethanol and subsequently through two xylene washes. Coverslips were mounted onto the slides using CytoSeal XYL mounting media (ThermoFisher). The slides were viewed and imaged using an Olympus BX40 microscope with an Olympus DP25 imaging system (Olympus, Center Valley, PA, USA). Serum alanine aminotransferase (ALT) concentrations were measured using a commercially available kit from Sigma-Aldrich with all assays and subsequent analyses performed according to the instructions provided by the manufacturer.

### Statistical Analyses

All statistical analyses were performed using Graphpad Prism software (Graphpad Software, La Jolla, CA, USA). Results were expressed as mean ± SEM. For data that passed normality tests, significance was established using the Student’s *t*-test when differences between two groups were analyzed, and analysis of variance when differences between three or more groups were compared followed by an appropriate *post hoc* test. If tests for normality failed, two groups were compared with a Mann-Whitney *U* test or a Kruskal-Wallis ranked analysis when more than two groups were analyzed. For the neurological score analyses, a two-way analysis of variance was performed followed by a Bonferroni multiple comparison *post hoc* test. Differences were considered significant for *p* values less than 0.05.

## Results

### Cortical Bile Acids Increase during AOM-Induced HE

**Figure 1:**
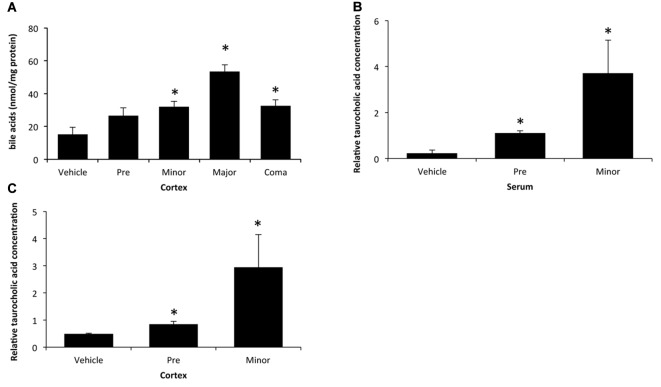
Bile acids are elevated in the cortex during azoxymethane (AOM)-induced hepatic encephalopathy (HE). **(A)** Concentrations of total bile acids in cortex homogenates during the indicated stages of neurological decline in AOM-treated mice that are normalized to protein concentrations. **(B)** Relative concentrations of taurocholic acid (TCA) in serum from vehicle and AOM-treated mice at the pre-neurological and minor neurological stages of AOM-induced HE. **(C)** Relative concentrations of TCA in cortex homogenates normalized by weight in vehicle and AOM-treated mice at the pre-neurological and minor neurological stages of AOM-induced HE. *p < 0.05 compared to vehicle-treated mice. *n* = 3 for total bile acid analyses and *n* = 5 for the metabolic screen for TCA.

As bile acids have been shown to be present in brain homogenates from patients with fulminant hepatic failure (Bron et al., 1977), bile acid concentrations were assessed throughout the time course of AOM-induced liver failure and HE. Levels of total cortical bile acids are increased as an early event during AOM-induced HE, with a significant increase beginning at a stage of minor neurological decline until coma when compared to vehicle-treated controls (Figure 1A). Due to this increase of bile acids occurring when minor neurological decline was present, a metabolic screen was performed in serum and cortex to identify metabolites that may contribute to the development of neurological complications in this model. While a variety of metabolites were dysregulated at stages of minor neurological decline compared to control, TCA was significantly elevated in both the serum (Figure 1B) and cortex (Figure 1C). Concentrations of the bile acids cholic acid, deoxycholic acid, taurochenodeoxycholic acid, taurodeoxycholic acid, tauroursodeoxycholic acid, α-muricholic acid and tauromuricholic acid were not significantly altered in the serum and tauromuricholic acid was not significantly increased in the cortex during minor neurological decline (data not shown). Together this identifies that total bile acids and the composition of the bile acid pool are dysregulated in the brain during AOM-induced HE.

### Cholestyramine Feeding Reduces Neuroinflammation

**Figure 2:**
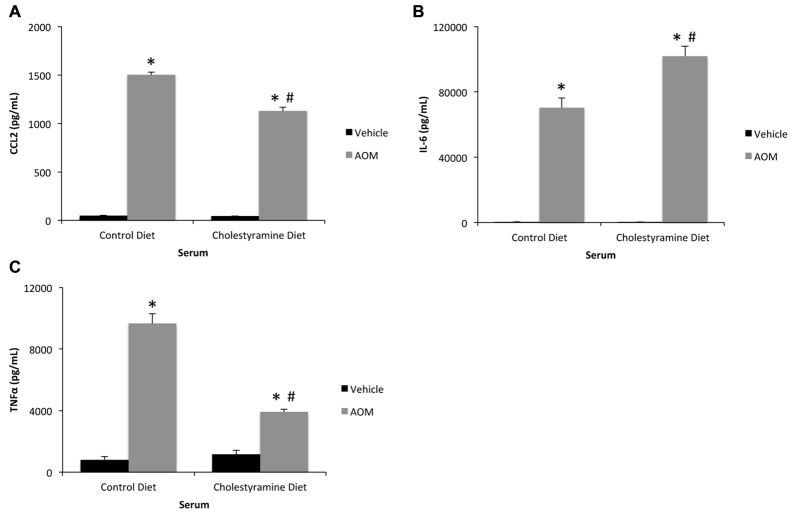
Systemic inflammation may be modulated by cholestyramine supplementation. **(A)** Serum consequence of chemokine ligand 2 (CCL2) concentrations measured by ELISA assay in control diet or cholestyramine diet-fed mice administered vehicle or AOM. **(B)** Interleukin-6 (IL-6) concentration in the serum of control diet or cholestyramine diet-fed mice injected with vehicle or AOM. **(C)** Serum tumor necrosis factor alpha (TNFα) concentrations measured by ELISA assay in control diet or cholestyramine diet-fed mice administered vehicle or AOM. *p < 0.05 compared to control diet vehicle-treated mice, ^#^*p* < 0.05 compared to control diet AOM-treated mice. *n* = 3 or greater for all analyses.

Previously we have identified that cholestyramine feeding reduces serum and cortical bile acid concentrations and subsequent neurological decline after AOM injection compared to control diet-fed mice (McMillin et al., 2016a). It is conceivable that the mechanism that led to the reduction of AOM-induced neurological decline after cholestyramine feeding was due to its ability to modulate systemic inflammation. While the levels of all cytokines remained significantly elevated in the serum of AOM-treated mice fed cholestyramine-enriched chow compared to vehicle-treated mice, levels of CCL2 and TNFα were significantly reduced while IL-6 was significantly increased in cholestyramine-fed AOM-treated mice compared to AOM-treated mice on a control diet (Figures 2A–C). Therefore cholestyramine does appear to have an effect on modulating systemic inflammation, though this appears to be dependent upon the cytokine measured.

**Figure 3:**
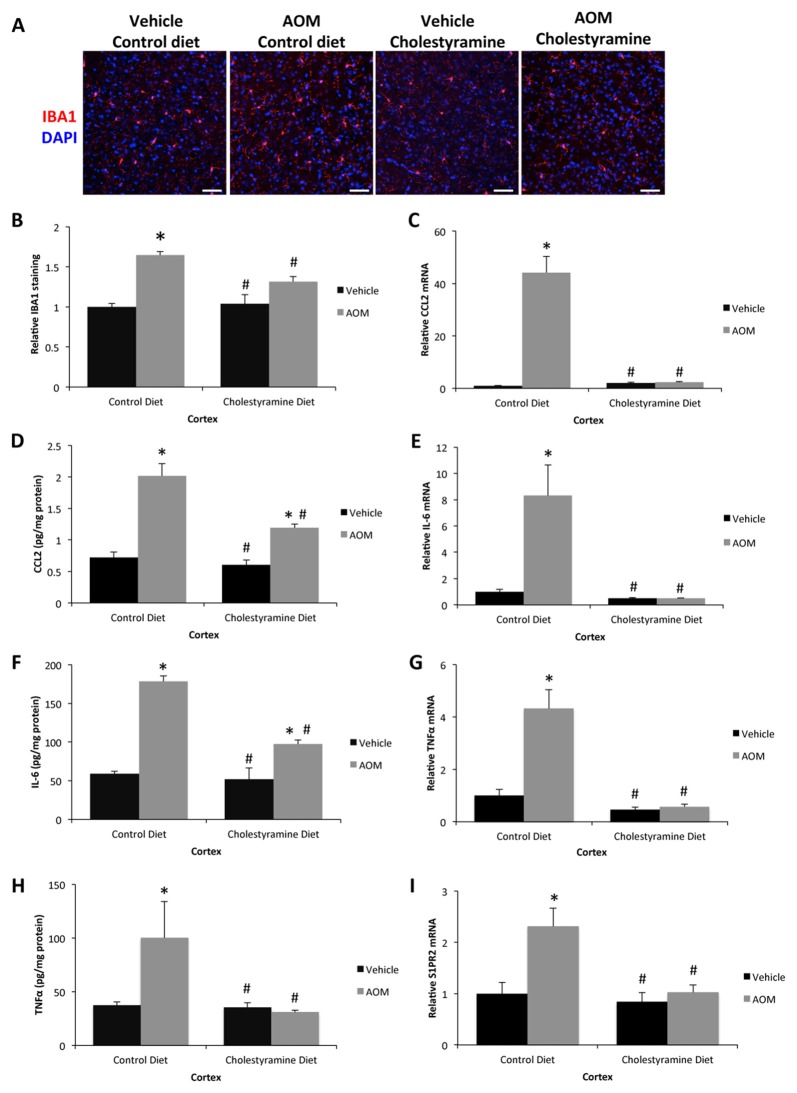
Neuroinflammation can be reduced by cholestyramine supplementation. **(A)** Representative staining for IBA1 (red) in the cortex from control diet and cholestyramine diet-fed mice administered saline (vehicle) or AOM. 4′,6-diamidino-2-phenylindole (DAPI; blue) was used to stain nuclei. The scale bar on the figure represents 50 μm. **(B)** Quantification of relative IBA1 field staining in the cortex from control diet and cholestyramine diet-fed mice administered vehicle or AOM. **(C)** Relative CCL2 mRNA expression in the cortex of control diet and cholestyramine diet-fed mice administered vehicle or AOM. **(D)** CCL2 concentrations in cortex homogenates normalized to total protein concentrations from control diet and cholestyramine diet-fed mice administered vehicle or AOM. **(E)** Relative IL-6 mRNA expression in the cortex of control diet and cholestyramine diet-fed mice administered vehicle or AOM. **(F)** IL-6 concentrations in cortex homogenates normalized to total protein concentrations from control diet and cholestyramine diet-fed mice administered vehicle or AOM. **(G)** Relative TNFα mRNA expression in the cortex of control diet and cholestyramine diet-fed mice administered vehicle or AOM. **(H)** TNFα concentrations in cortex homogenates normalized to total protein concentrations from control diet and cholestyramine diet-fed mice administered vehicle or AOM. **(I)** Relative cortex sphingosine-1-phosphate receptor 2 (S1PR2) mRNA expression in control diet and cholestyramine diet-fed mice administered vehicle or AOM. *p < 0.05 compared to control diet vehicle-treated mice, ^#^*p* < 0.05 compared to control diet AOM-treated mice. *n* = 3 or greater for all analyses.

In regards to bile-acid mediated signaling, FXR signaling contributed in part to the neurological decline observed during AOM-induced HE, though FXR signaling does not account for the exacerbated neuroinflammatory response observed during this disease state (data not shown). Neuroinflammation is a known pathological contributor to HE and significant microglia proliferation, as assessed by IBA1 staining, was observed in control diet-fed AOM-treated mice compared to cholestyramine diet-fed mice administered AOM or vehicle-treated mice on either diet (Figures 3A,B). Microglia activation and proliferation can occur in this model due to increased CCL2 secretion from neurons that acts on microglia (McMillin et al., 2014a). There was a significant increase of CCL2 expression in control diet-fed AOM-treated mice but this increase was significantly reduced in cholestyramine-fed AOM-treated mice as shown by mRNA (Figure 3C) and protein expression (Figure 3D). In addition to this, expression of IL-6 was significantly increased in control diet-fed AOM-treated mice but was significantly lessened in cholestyramine-fed AOM-treated mice as shown by mRNA (Figure 3E) and protein expression analyses (Figure 3F). TNFα mRNA (Figure 3G) and protein (Figure 3H) expression were significantly increased in control diet-fed AOM-treated mice but was significantly reduced in cholestyramine-fed AOM-treated mice. Cholestyramine-fed AOM-treated mice had reduced levels of S1PR2 mRNA in the cortex compared to control diet-fed AOM-treated mice, supporting a role for total bile acids regulating S1PR2 expression in the brain (Figure 3I).

### Primary Neurons Increase CCL2 Expression via S1PR2-Mediated Mechanisms

**Figure 4:**
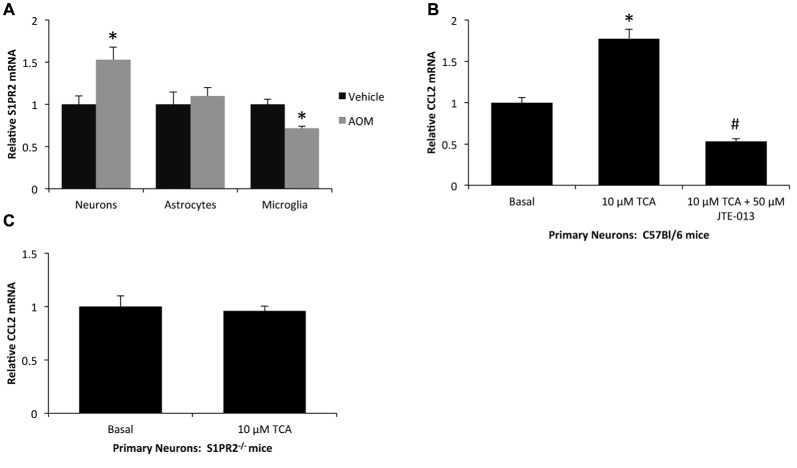
S1PR2 mediates CCL2 expression in primary neurons. **(A)** Relative S1PR2 mRNA expression in neurons, astrocytes and microglia isolated from vehicle and AOM-treated mice. **(B)** Relative CCL2 mRNA expression in primary neurons isolated from C57Bl/6 mice that were treated with 10 μM TCA and/or 50 μM JTE-013. **(C)** Relative CCL2 mRNA expression in primary neurons isolated from S1PR2^−/−^ mice that were treated with 10 μM TCA. *p < 0.05 compared to basal, ^#^*p* < 0.05 compared to 10 μM TCA-treated primary neurons. *n* = 4 for isolated cell S1PR2 analyses and *n* = 3 for primary neuron S1PR2 mRNA analyses.

In order to determine the cellular localization and expression of S1PR2, neurons, astrocytes and microglia were isolated from vehicle and AOM-treated mice and were assessed for S1PR2 mRNA expression. There was a significant increase of S1PR2 mRNA in neurons isolated from AOM-treated mice compared to neurons isolated from vehicle-treated controls, while mRNA levels were static in astrocytes and decreased in microglia following AOM administration (Figure 4A). As neurons had a significant increase in S1PR2 expression during AOM-induced HE, and we have previously demonstrated that neuron-derived CCL2 expression contributes to the neuroinflammation observed during HE (McMillin et al., 2014a), the effects of TCA on neuronal CCL2 expression were assessed. Treatment of primary mouse neurons with 10 μM TCA was found to significantly increase CCL2 expression which was attenuated if the cells were co-treated with the S1PR2 antagonist JTE-013 (Figure 4B). In contrast, TCA treatment had no effect on CCL2 mRNA expression in primary neurons isolated from S1PR2^−/−^ mice (Figure 4C).

### S1PR2-Signaling Promotes AOM-Induced Neurological Decline

**Figure 5:**
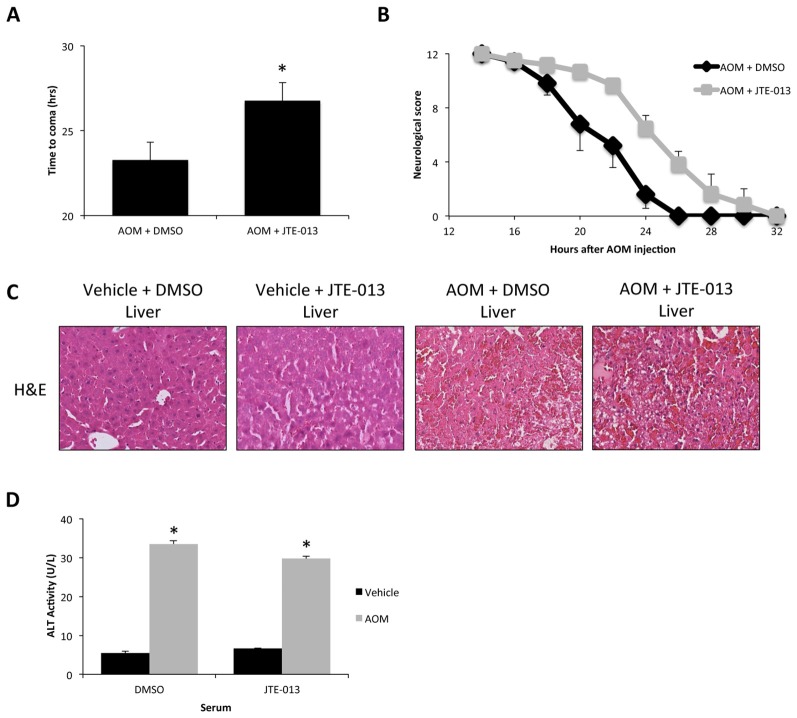
JTE-013 infusion reduces neurological decline without influencing hepatic injury in AOM-treated mice. **(A)** Time taken to progress to coma in hours of AOM-treated mice infused with DMSO or JTE-013. **(B)** Neurological score analyses as assessed by reflex scores and ataxia at the indicated hours post AOM injection in AOM-treated mice infused with DMSO or JTE-013. **(C)** Representative H&E images from the livers of vehicle or AOM-treated mice infused with DMSO or JTE-013. **(D)** Alanine aminotransferase (ALT) activity in the serum of vehicle or AOM-treated mice infused with DMSO or JTE-013. *p < 0.05 compared to AOM + DMSO (for time to coma analyses) or vehicle-treated mice infused with DMSO (for serum ALT activity analyses). *n* = 6 for time to coma and neurological score analyses and *n* = 4 for serum ALT analyses.

In order to see the effects of S1PR2 signaling during AOM-induced HE, mice were implanted with osmotic minipumps to infuse JTE-013 directly into the lateral ventricle of the brain prior to vehicle or AOM injection. JTE-013 infusion was found to significantly lengthen the time taken to reach coma in AOM-treated mice compared to 50% DMSO-infused controls (Figure 5A). Neurological decline of AOM-treated mice began earlier and at a greater rate than AOM-treated mice infused with JTE-013 with a significant difference between the two groups (*p* = 0.0381; Figure 5B). As AOM-induced HE is a model of liver failure, overall liver histology and function were assessed to determine if the effect of JTE-013 could be due to an improvement in liver pathology rather than a direct protective action on the brain. No difference in liver damage was observed between vehicle and JTE-013-infused mice after saline or AOM injection as observed by H&E histochemistry (Figure 5C) or serum ALT concentrations (Figure 5D).

### JTE-013 Infusion Reduces AOM-Induced Neuroinflammation

**Figure 6:**
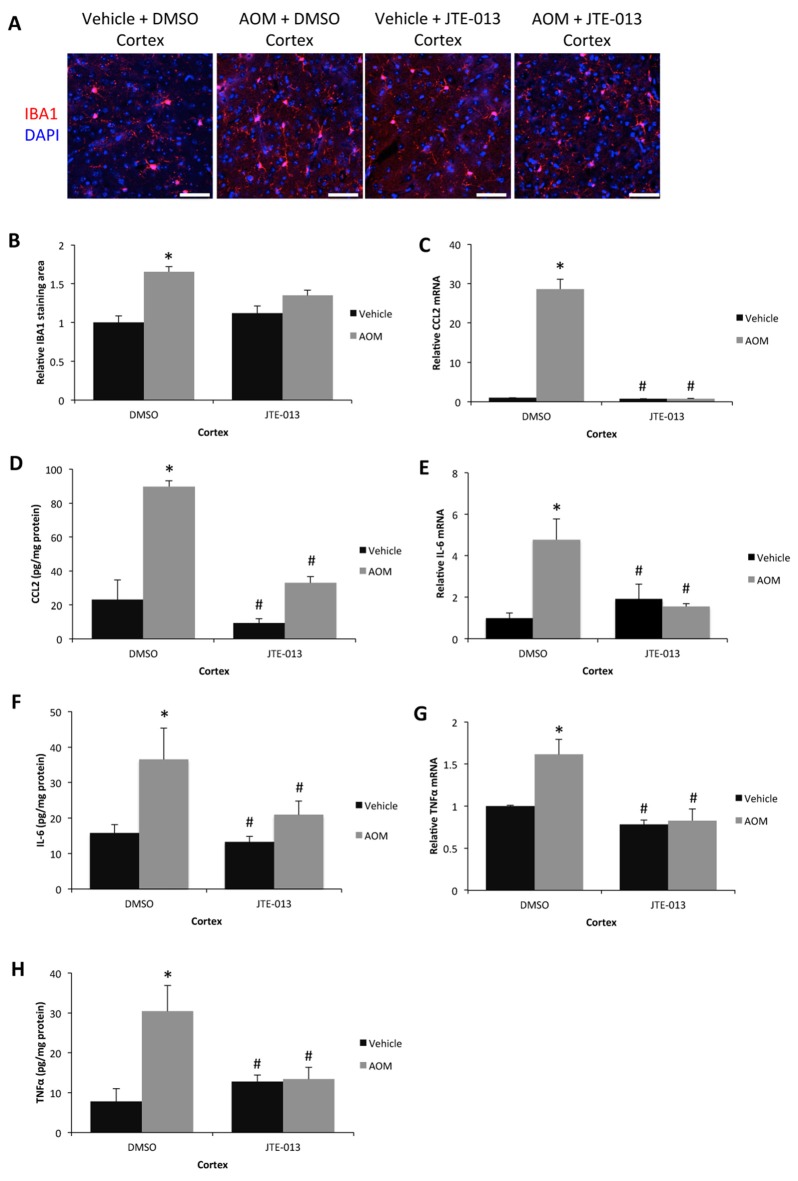
Neuroinflammation can be reduced by JTE-013 infusion. **(A)** Representative staining for IBA1 (red) in the cortex from DMSO or JTE-013 infused mice administered saline (vehicle) or AOM. DAPI (blue) was used to stain nuclei. The scale bar on the figure represents 50 μm. **(B)** Quantification of relative IBA1 field staining in the cortex from DMSO or JTE-013-infused mice administered vehicle or AOM. **(C)** Relative CCL2 mRNA expression in the cortex from DMSO or JTE-013-infused mice administered vehicle or AOM. **(D)** CCL2 concentrations in cortex homogenates normalized to total protein concentrations from DMSO or JTE-013-infused mice administered vehicle or AOM. **(E)** Relative cortex IL-6 mRNA expression in DMSO or JTE-013-infused mice administered vehicle or AOM. **(F)** Cortex IL-6 concentrations in homogenates normalized to total protein concentrations from DMSO or JTE-013-infused mice administered vehicle or AOM. **(G)** Relative TNFα mRNA expression in the cortex from DMSO or JTE-013-infused mice administered vehicle or AOM. **(H)** TNFα concentrations in cortex homogenates normalized to total protein concentrations from DMSO or JTE-013-infused mice administered vehicle or AOM. *p < 0.05 compared to DMSO-infused vehicle-treated mice, ^#^*p* < 0.05 compared to DMSO-infused AOM-treated mice. *n* = 3 or greater for all analyses.

Due to the protective effect of JTE-013 on AOM-induced HE without a concurrent improvement in liver pathology suggests that the protective mechanism of JTE-013 infusion is primarily in the brain. As inflammation is a core component of HE, neuroinflammation was assessed in JTE-013-infused mice. The significant increase in number of microglia observed during AOM-induced HE was not observed in AOM-treated mice infused with JTE-013 (Figures 6A,B). CCL2 mRNA expression (Figure 6C) and protein expression (Figure 6D) in the cortex were significantly increased in AOM-treated mice infused with 50% DMSO and this increase in expression was not present in AOM-treated mice infused with JTE-013. IL-6 mRNA (Figure 6E) and protein (Figure 6F) expression were significantly increased in the cortex of AOM-treated mice infused with 50% DMSO and were significantly reduced in vehicle-treated mice or AOM-treated mice infused with JTE-013. The mRNA and protein expression of TNFα was elevated in AOM-treated mice infused with 50% DMSO compared to vehicle-treated mice or AOM-treated mice infused with JTE-013 (Figures 6G,H). Taken together, these data support that the inhibition of S1PR2 by JTE-013 infusion reduces microglia proliferation and neuroinflammation during AOM-induced HE.

## Discussion

**Figure 7:**
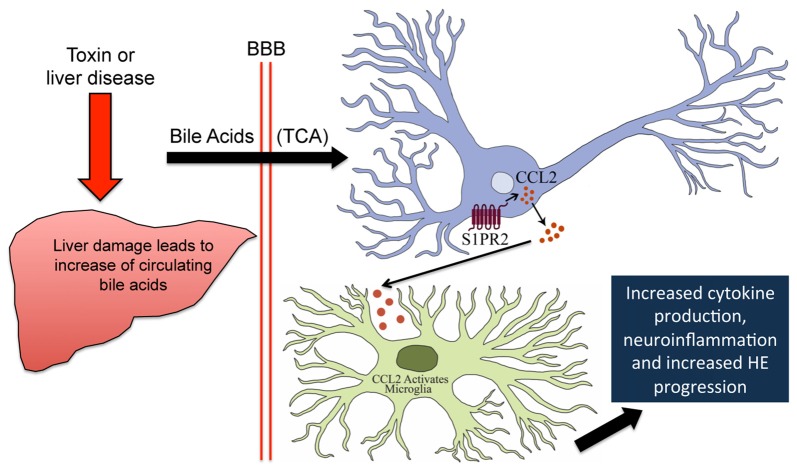
Working model of S1PR2-mediated neuroinflammation during AOM-induced HE. AOM-induced liver failure disrupts the enterohepatic circulation and causes hepatocyte death leading to an increase of circulating bile acids including TCA. TCA crosses the leaky blood brain barrier and binds S1PR2 in neurons. This leads to increased expression and secretion of CCL2 from neurons, which binds receptors on microglia leading to their activation. This ultimately results in increased proinflammatory cytokine expression and worse HE outcomes.

The major findings from this study relate to the role that bile acid-mediated S1PR2 signaling plays in neurological decline, microglia activation and subsequent neuroinflammation that occur during HE. These data support that total bile acids, and TCA in particular, are elevated as an early event during AOM-induced HE. Microglia activation, and subsequent upregulation of proinflammatory cytokines, occurs during AOM-induced HE and prior feeding of a cholestyramine-supplemented diet reduced their activation. S1PR2 is upregulated in neurons isolated from AOM-treated mice and TCA is able to induce CCL2 mRNA expression via S1PR2-mediated signaling. A working model of these findings is shown which suggests that increased neural bile acids during HE activate S1PR2, which leads to microglia activation, neuroinflammation and exacerbated neurological decline (Figure 7). Thus, reducing levels of bile acids or antagonizing S1PR2 activity may be potential treatment modalities for the management of HE due to acute liver failure.

The elevation of bile acids during HE was first identified by Bron et al. (1977) in a report that detailed that bile acids were elevated in the cerebrospinal fluid and brain tissue in patients with fulminant hepatic failure. At that time, in the absence of known specific bile acid receptors, it was assumed that the elevations were not high enough to cause a direct effect on cerebral edema and bile acids were not investigated during HE for many years. We now understand that bile acids have the capability to signal through a variety of receptors including the G-protein coupled receptors TGR5, S1PR2, α5β1 integrin and the nuclear receptors FXR, VDR, PXR, GR and CAR, indicating that small changes in their concentrations can influence numerous cell signaling pathways (McMillin et al., 2016b). The elevation of bile acids observed in this study suggest that this could be contributing to neurological decline. We previously identified that feeding a cholestyramine-supplemented diet to AOM-treated mice reduced FXR-mediated signaling in neurons and improved neurological outcomes (McMillin et al., 2016a) however, this pathway could not adequately account for the increased neuroinflammation observed during HE. In addition, activation of TGR5 via an ICV infusion of betulinic acid during AOM-induced HE was found to reduce both neuroinflammation and neurological decline (McMillin M. et al., 2015). In order to better classify specific signaling pathways that are influenced during HE, we performed a metabolic screen and identified that TCA was increased in the serum and brain following AOM injection in mice. While TCA has not been studied in the context of neuroinflammation, it has been shown to increase the expression of proinflammatory genes in hepatocytes (Allen et al., 2011), supporting that this bile acid may promote microglia activation during HE. Because TCA generates its effects primarily through S1PR2 (Studer et al., 2012), this study focused on S1PR2-mediated signaling and its role in HE. That being said, one caveat of this study is that the metabolic panel only measured seven bile acids in the serum and two in the cortex identifying that more expansive studies are necessary to fully characterize the activity of individual bile acids and their signaling pathways on the pathogenesis of HE.

S1PR2 has been previously described to be critical for normal neurological function as S1PR2-knockout mice develop normally but begin to have spontaneous and sporadic seizures due to neuronal hyperexcitability (MacLennan et al., 2001). Based upon this report, it is conceivable that increased S1PR2 activity could be responsible for some of the neurological dysfunction observed during HE. Cholestyramine-fed mice administered AOM have reduced neurological decline and reduced S1PR2 mRNA expression indicating that bile acids control transcriptional activity of S1PR2 and that there is a correlation between neurological decline and S1PR2 expression. In order to test the direct role of S1PR2 in HE progression this model, JTE-013 was infused directly into the brain prior to AOM injection and was found to improve neurological outcomes without influencing liver damage or pathology. S1PR2 mRNA was detected in isolated neurons, astrocytes and microglia though its expression was only increased in neurons during AOM-induced HE. Due to this, primary neurons were used for the *in vitro* experiments in this study, though S1PR2-mediated signaling could influence other cells as well and these cell populations will need to be investigated in future studies.

Most of the current therapies for HE, such as lactulose and rifaxamin, are targeted at reducing levels of systemic and brain ammonia by reducing its absorption from the intestinal lumen though clinical outcomes often are poor either due to a lack of response to the medications or failure to follow treatment regimens (Wijdicks, 2016). Ammonia may generate effects besides inducing astrocyte swelling and metabolic dysfunction as it has been shown to activate microglia in a rat cell culture model indicating that ammonia and neuroinflammation may work in tandem to promote pathology during this disease state (Zemtsova et al., 2011). It is worth noting that most of the contributors to neuroinflammation during HE are unknown. The current study was the first to identify bile acids as inducers of microglia activation during HE. Cholestyramine-supplementation of mice prior to AOM-injection was found to reduce AOM-induced neuroinflammation by inhibiting microglia activation and reducing the expression of the proinflammatory mediators CCL2, IL-6 and TNFα in the cortex. In the serum of cholestyramine-supplemented AOM-treated mice, levels of CCL2 and TNFα were decreased though IL-6 increased compared to AOM-treated mice with the control diet. This differential observation between the serum and cortex indicates that cholestyramine generates its consequences on neuroinflammation in a manner different from its effects on systemic inflammation. Furthermore, recent studies have shown that the liver damage as a result of acetaminophen toxicity was exacerbated in mice fed a cholestyramine-enriched diet (Bhushan et al., 2013), therefore it is conceivable that the cholestyamine-induced increase in IL-6 may be derived from the liver in our model. As TCA was found to be elevated as an early event in this model, it was not surprising that similar findings were observed in AOM-treated mice infused with JTE-013. To ensure that these effects were truly S1PR2-dependent, primary neurons were treated with TCA, which induced CCL2 mRNA expression. This effect was reversed by JTE-013 treatment or was absent from neurons isolated from S1PR2^−/−^ mice. S1PR2 has been shown to promote inflammation in models outside the brain as JTE-013 treatment prior to antigen treatment in a mouse model of allergic inflammation reduced immune cell infiltration and CCL2 expression (Oskeritzian et al., 2015). Other studies employing S1PR2 knockout mice have identified that S1PR2-mediated signaling is required for both vascular permeability and inflammation during endotoxemia (Zhang et al., 2013).

There is currently a paucity of studies investigating neuroinflammation and S1PR2 and this study is the first to examine this pathway during a metabolic brain disorder. That being said, it is possible that S1PR2-mediated signaling could influence aspects of HE pathology besides neuroinflammation that were not investigated in this report. For example, S1PR2 expression has been reported in brain endothelial cells and its activity increased MMP-9 activity and promoted blood-brain barrier permeability during stroke (Kim et al., 2015). AOM-induced HE is associated with increased blood-brain barrier permeability and an increase of MMP-9 expression and we have previously demonstrated that certain bile acids can increase the permeability of the blood brain barrier (Quinn et al., 2014) indicating that bile acid-mediated S1PR2 signaling could conceivably influence this aspect of pathology during HE (Chastre et al., 2014; McMillin M. A. et al., 2015). Furthermore, S1PR2 has the capability to influence FXR signaling, which has been previously identified to promote HE pathogenesis (McMillin et al., 2016a). Deletion of FXR in mice has been shown to lead to increased serum concentrations of TCA and other conjugated bile acids that signal through S1PR2, and these mice have impaired memory and reduced motor coordination (Huang et al., 2015). S1PR2-mediated signaling also increases the expression of SHP, which binds to FXR and is known to downregulate numerous genes including the bile acid synthesis enzyme CYP7A1 (Calkin and Tontonoz, 2012; Studer et al., 2012). S1PR2 signaling has also been shown to have effects on endothelial cell function during hyperglycemia (Chen et al., 2015; Liu et al., 2016) and while AOM-induced liver failure generally leads to reduced blood glucose levels (Matkowskyj et al., 1999), which was controlled through dextrose injection in this study, the crosstalk between altered blood glucose levels and S1PR2-mediated effects were not investigated. More studies are necessary to fully classify the effects of S1PR2-mediated signaling on pathology, metabolism and other signaling pathways during HE.

These data represent the first demonstration of a role in bile acid-mediated S1PR2 signaling in both neuroinflammation and neurological decline associated with HE. Taken together with previous studies, these findings support the hypothesis that following liver failure, bile acids are elevated in the circulation, gain entry into the brain and bind S1PR2 on neurons, which secrete CCL2 and lead to the activation of microglia. This ultimately leads to the release of proinflammatory cytokines from microglia, promoting the neuroinflammatory state that contributes to the progression of HE. Ultimately, this report identifies S1PR2 as a potential therapeutic target for the management of neuroinflammation and neurological dysfunction during HE.

## Author Contributions

MM, GF, SG, SK, JD, AP, BJ, JK, AW and SD helped acquire and analyze data; critically edited and approved final version of manuscript; agree to be accountable for all aspects of the work in ensuring that questions related to the accuracy or integrity of any part of the work are appropriately investigated and resolved. MM and SD conceived the work; drafted the manuscript.

## Conflict of Interest Statement

The authors declare that the research was conducted in the absence of any commercial or financial relationships that could be construed as a potential conflict of interest.
